# Study of Class 1, 2, and 3 Integrons, Antibiotic Resistance Patterns, and Biofilm Formation in Clinical *Staphylococcus aureus* Isolates from Hospital-Acquired Infections

**DOI:** 10.3390/pathogens14070705

**Published:** 2025-07-17

**Authors:** Eman E. Hegazy, Wageih Salem ElNaghy, Marwa M. Shalaby, Sarah M. Shoeib, Nashwa S. M. Abdeen, Mohamed H. Fouda, Ola A. Elshora, Mohammed H. Elnaggar, Waleed Elrefaey, Rasha Youssef Hagag, Ahmed A. Elhadidy, Mohamed A. Elsebaey, Mohamed A. Eltomey, Ahmed Mohamed El Nakib, Mai Nabil Ageez, Maha S. Elnady

**Affiliations:** 1 Department of Medical Microbiology and Immunology, Faculty of Medicine, Tanta University, Tanta 31527, Egypt; marwa.shalabi@med.tanta.edu.eg (M.M.S.); maha.elnady@med.tanta.edu.eg (M.S.E.); 2 Department of Clinical Pathology, Faculty of Medicine, Tanta University, Tanta 31527, Egypt; sara.shoeib@med.tanta.edu.eg (S.M.S.); nashwa.sallam@med.tanta.edu.eg (N.S.M.A.); mohamed.fouda@med.tanta.edu.eg (M.H.F.); ola.a.elshora@gmail.com (O.A.E.); 3 Department of Internal Medicine, Faculty of Medicine, Tanta University, Tanta 31527, Egypt; mohamed.elnagar1@med.tanta.edu.eg (M.H.E.); dr.waleedelrefaey@gmail.com (W.E.); rasha.hagag@med.tanta.edu.eg (R.Y.H.); ahmed.elhadedy@med.tanta.edu.eg (A.A.E.); mohamedelsebaey79@gmail.com (M.A.E.); 4 Department of Radiology and Imaging, Faculty of Medicine, Tanta University, Tanta 31527, Egypt; mohamed.eltomey@med.tanta.edu.eg; 5 Department of Tropical Medicine, Faculty of Medicine, Mansoura University, Mansoura 35511, Egypt; el_naqueeb@mans.edu.eg; 6 Department of Obstetrics and Gynecology, Faculty of Medicine, Tanta University, Tanta 31527, Egypt; mai.ageez@med.tanta.edu.eg

**Keywords:** *Staphylococcus aureus*, methicillin-resistant, integrons, biofilm, antibiotic resistance

## Abstract

Antibiotic resistance and biofilm formation complicate *Staphylococcus aureus* infections, raising concerns for global health. Understanding antimicrobial resistance and biofilm formation in these pathogens is essential for effective infection management. The current research aimed to assess antibiotic resistance patterns, biofilm formation, and the occurrence of integron classes 1, 2, and 3 in clinical *S. aureus* isolates. The disc diffusion method tested antibiotic susceptibility. MRSA strains were identified by cefoxitin disc diffusion, and the *mecA* gene by PCR. The D-test also assessed macrolide–lincosamide–streptogramin B. A microtiter plate assay assessed biofilm formation. By PCR, integron classes were examined. Of the 63 *S. aureus* isolates, 25 were MSSA and 38 were MRSA. Pus (39.5%) was the most prevalent clinical source of MRSA isolates, while blood (24%) was the predominant source of MSSA isolates. MRSA isolates were more resistant to clindamycin, ciprofloxacin, ofloxacin, levofloxacin, tetracycline, and doxycycline than MSSA isolates. In total, 76.2% of the isolates produced biofilm. Biofilm-producing isolates were more resistant to cefoxitin and clindamycin. The isolates had 33.3% cMLSB resistance. The *intI1* gene was found in 21 *S. aureus* isolates (33.3%), whereas the *intI2* or *intI3* genes were not detected. Our findings demonstrate the need for strict infection control to prevent the spread of resistant bacteria.

## 1. Introduction

*Staphylococcus aureus (S. aureus)* is a common human colonizer of skin and mucosal membranes. It is an opportunistic pathogen and a significant human pathogen capable of causing various infections, including superficial and systemic diseases, which lead to exceptionally high morbidity and mortality rates [1,2].

As one of the ESKAPE pathogens *(Enterococcus faecium*, *Staphylococcus aureus, Klebsiella pneumoniae*, *Acinetobacter baumannii*, *Pseudomonas aeruginosa*, *and Enterobacter species)*, these organisms are known for their multidrug resistance and association with nosocomial infections worldwide [3]. *S. aureus* has developed resistance to many antibiotics, like aminoglycosides, β-lactams, and macrolide–lincosamide–streptogramin B, either through chromosomal mutations or by acquisition of resistance determinant from other bacteria. Consequently, the World Health Organization (WHO) has listed *S. aureus* as a high-priority antimicrobial-resistant pathogen [4,5].

Methicillin-resistant *S. aureus* (MRSA) is a strain of *S. aureus* that exhibits resistance to beta-lactam antibiotics. Its multidrug resistant phenotype reduces treatment alternatives and increases human susceptibility to severe infections [6]. This resistance is attributed to target modification mediated by the *mecA* or *mecC* genes found within a mobile genetic element called staphylococcal cassette chromosome mec (SCCmec). These genes encode penicillin-binding protein 2a (PBP2a), which has low affinity for β-lactam antibiotics [7].

The mecA gene can be acquired by mobile genetic elements (MGEs) that can transfer around inside a genome or move from one species to another [8]. MGEs, such as plasmids, transposons, and integrons, are responsible for the acquisition and dissemination of antimicrobial resistance genes through horizontal gene transfer [9]. Four classes categorize integrons, and antimicrobial resistance has been associated with integron classes 1–3 [10]. Among these, class 1 and class 2 integrons are the most predominant and have been identified in both Gram-negative and Gram-positive bacteria, including *S. aureus* [11].

Multidrug-resistant (MDR) *S. aureus* strains that can form biofilms contribute substantially to treatment failure and the persistence of infections. Biofilms provide a protective shield for the bacteria against antibiotics and the immune responses of the host [12].

Biofilms are complex microbial communities that adhere to surfaces and are surrounded in an extracellular matrix that facilitates horizontal gene transfer of resistance determinants [13]. MDR *S. aureus* strains with biofilm-forming capacity are particularly associated with severe clinical infections [14].

Integrons have been thoroughly studied in Gram-negative bacteria, but recent studies have begun to emphasize their significance in Gram-positive species, especially *S. aureus* [15]. The presence of integrons may be associated with increased antibiotic resistance and an enhanced capacity for biofilm formation in *S. aureus*. Our objective was to evaluate antibiotic resistance patterns, biofilm formation, and the presence of integron classes 1, 2, and 3 in clinical isolates of *S. aureus*.

## 2. Materials and Methods

### 2.1. Study Design and Sample Collection

This hospital-based cross-sectional study investigated 63 non-repetitive *Staphylococcus aureus* isolates obtained from patients admitted to different intensive care units and clinical departments at Tanta University Hospitals. Various consecutive clinical samples were aseptically collected from hospitalized patients showing signs and symptoms of hospital-acquired infections, according to the infection site, over the course of eight months, from October 2024 to May 2025, including respiratory samples (sputum, endotracheal tube [ETT] aspirates), blood, urine, and pus. The pus was from surgical wound infections (post-caesarean sections, myomectomy, hysterectomy) and burn infections. All samples were transferred immediately to the Medical Microbiology Department laboratory for processing.

### 2.2. Isolation and Identification of S. aureus

All clinical samples were cultivated on blood agar, nutrient agar, and mannitol salt agar (Oxoid, Hampshire, UK), then incubated aerobically at 37 °C for 24–48 h. Blood samples were processed using the BacT/ALERT 3D 60 automated culture system (bioMérieux, Marcy-l’Étoile, France). Standard biochemical tests were employed for identifying all *S. aureus* isolates [16]. The identification of species was verified using an automatic VITEK2 system for Gram-positive identification (bioMérieux, Marcy-l’Étoile, France) in line with the manufacturer’s guidelines. All confirmed *S. aureus* isolates were preserved at −80 °C in brain heart infusion broth supplemented with 20% glycerol (Merck, Darmstadt, Germany) for evaluation of antibiotic susceptibility testing, biofilm formation, and molecular study. For bacterial revival, one loopful was streaked over blood agar and incubated aerobically for 18–24 h at 37 °C.

### 2.3. Antimicrobial Susceptibility Testing

**Figure 1:**
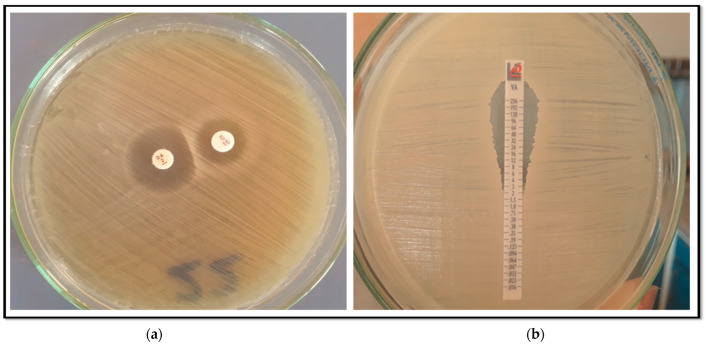
(**a**) D zone test for inducible clindamycin resistance; (**b**) Vancomycin E-test showing a sensitive isolate of *S. aureus* (MIC = 2 µg/mL).

The Kirby–Bauer disc diffusion method, recommended by CLSI 2025 [17], was used to assess the susceptibility of *S. aureus* isolates on Mueller–Hinton agar plates (HiMedia, Mumbai, India) to the following antibiotics: erythromycin (15 µg), cefoxitin (30 µg), clindamycin (2 µg), ofloxacin, ciprofloxacin. (5 µg), doxycycline, tetracycline (30 µg), linezolid (30 µg), gentamicin (10 µg), and trimethoprim/sulfamethoxazole (1.25/23.75 µg) (HiMedia, Mumbai, India). Vancomycin susceptibility was determined by minimum inhibitory concentration (MIC) testing using E test strips (bioMérieux, Marcy-l’Étoile, France) according to the manufacturer’s instructions, with CLSI-defined breakpoints: sensitive (≤2 µg/mL) as shown in (Figure 1), intermediate (4–8 µg/mL), and resistant (≥16 µg/mL). MRSA isolates were identified by evaluating their resistance to cefoxitin (FOX, 30 µg) disc. Isolates exhibiting an inhibition zone of less than 21 were classified as MRSA [17]. The *S. aureus* ATCC 25923 strain was used as quality control.

### 2.4. Phenotypic Detection of Inducible Clindamycin Resistance

To evaluate if the erythromycin resistant isolates exhibited inducible clindamycin resistance, the D-zone test described in CLSI guidelines [17] was applied. On Mueller–Hinton agar plates inoculated with 0.5 McFarland fresh bacterial suspensions, an erythromycin (15 µg) disc was positioned 15 mm (edge to edge) from the clindamycin (2 µg). After 18 h of incubation at 37 °C, the plates were inspected. The inhibitory zone sizes were interpreted according to the following: flattening the sensitive zone of inhibition to clindamycin (known as the D-zone) near the erythromycin disc, indicative of a positive result for inducible clindamycin resistance (iMLSB phenotype) as shown in (Figure 1). The isolate exhibited erythromycin resistance and clindamycin sensitivity, characterized by circular zones of inhibition, indicative of the MS phenotype. Strains exhibiting resistance to both antibiotics were considered to possess constitutive macrolide–lincosamide–Streptogramin B resistance (cMLSB phenotype).

### 2.5. Biofilm Formation by Tissue Culture Plate Technique

*Staphylococcus aureus* isolates were evaluated for biofilm formation as previously explained [18]. Each isolate was first cultured overnight in Trypticase soy broth (HiMedia, Mumbai, India) tryptic soy broth. We adjusted the bacterial suspensions to match a standard turbidity of 0.5 McFarland, then a volume of 100 μL from each suspension was transferred into the wells of a sterile 96-well microtiter plate except for the last column, which is used as a negative control. Then we incubated the plate for 24 h. Following incubation, the wells were carefully evacuated and rinsed with saline to eliminate any non-adherent cells. Biofilm visualization was achieved by staining the wells with 150 μL of 0.2% crystal violet for 15 min at room temperature. We discarded the excess stain and then the wells were cleaned with water. After air drying at room temperature, the retained crystal violet was solubilized with 95% ethanol. The assay was performed in triplicate. An ELISA reader recorded the optical density (OD) of every well at 620 nm. The mean OD values were determined for all examined isolates and negative controls. Based on previously defined standards, the isolates were classified into four groups: non biofilm producers (ODs ≤ ODc) and weak (ODc ≤ ODs ≤ 2× ODc), moderate (2× ODc ≤ ODs ≤ 4× ODc), and strong biofilm producers (ODs > 4× ODc) [19]. ODc describes the OD of the negative control, and ODs describe the OD of the experimental samples.

### 2.6. Molecular Detection of MRSA and Three Integron Classes by PCR

**Figure 2:**
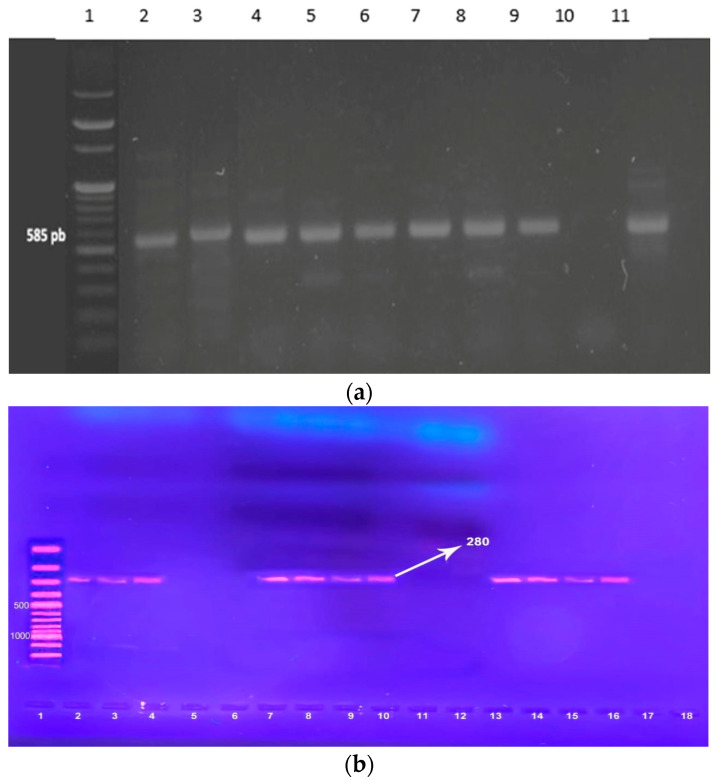
(**a**) Gel electrophoresis of PCR amplification of *the mecA* gene. Lane 1: 100 pb DNA ladder marker. Lane 2: mecA-positive control (585 bp). Lane 3, 4, 5, 6, 7, 8, 9, 11 = mecA-positive isolates Lane 10 = mecA-negative isolate. (**b**). Agarose gel electrophoresis of PCR products of the *intI1* gene amplicon among *S. aureus*, positive isolates gave a single band of 280 bp. Lane 1: 100 pb DNA ladder marker Lane 2: *intI1* -positive control (280 bp). Lane 3, 4, 7, 8, 9, 10, 13, 14, 15, 16 = *intI1*-positive isolates Lane 5, 6, 11, 12, 17, 18 = *intI1*-negative isolate.

MRSA isolates based on phenotypic testing (resistant to cefoxitin) have been verified by amplifying the *mecA* gene. Additionally, all *S. aureus* isolates underwent amplification of *intI1*, *intI2*, and *intI3*. DNA was extracted using the QIAamp DNA Mini Kit (Qiagen, Hilden, Germany) according to the manufacturer’s instructions. Amplification was performed in accordance with the procedure described by Zhang et al. and Zomorodi et al. [20,21]. The *S. aureus* ATCC 25923 strain served as a positive control, while distilled water was utilized as a negative control in substitute of the DNA template. The PCR products were visualized and photographed under UV light following electrophoresis for 45 min at 100 V through a 1% agarose that contained ethidium bromide (1 μg/mL) (Figure 2). The utilized primers, the amplicon sizes, and the thermal cycling program are included in Table S1 in the Supplementary Materials.

### 2.7. Statistical Analysis of the Data

The IBM SPSS 20.0 software (IBM Corp., Armonk, NY, USA, 2011) processed the data entered into the computer. 2011 (IBM Corp, Armonk, NY, USA). Category data were expressed as numbers and percentages. The two groups were compared using a chi-squared test. The Fisher exact test or Monte Carlo adjustment was employed when more than 20% of expected cells counted less than 5. The Kolmogorov–Smirnov test evaluated continuous data normality. Range, mean, standard deviation, median, and interquartile range were used for quantitative data. To compare two normally distributed quantitative data sets, Student’s *t*-test was used. Result significance was determined at the 5% level. The crude odds ratio (COR) and 95% confidence interval (CI) were computed. Significant variables associated with biofilm production, including type of specimen, *mec A*, and MLSB phenotypes, were entered into backward Wald binary logistic regression models, with the adjusted odds ratio (AOR) and 95% CI used to investigate the independent determinants of biofilm production. No multicollinearity was detected.

## 3. Results

### 3.1. Demographic Data and Clinical Source of MRSA and MSSA Isolates

The basic patient characteristics for isolated MRSA and MSSA are presented in Table 1. We evaluated 63 non-repetitive clinical *S. aureus* isolates, including 38 (60.3%) MRSA and 25 (39.7%) MSSA isolates from various hospital-acquired infections. The mean age of patients with MRSA was 41.47 ± 14.62 years, whereas those with MSSA were significantly older (49.24 ± 11.68 years, *p* = 0.030). No statistically significant difference in gender distribution was detected between MRSA and MSSA cases. The most frequent clinical source of MRSA isolates was pus (39.5%), whereas blood (24%) was the predominant source for MSSA isolates.

### 3.2. Antimicrobial Resistance Patterns Among MRSA and MSSA

The resistance patterns of 63 *S. aureus* isolates to various antimicrobials and the differences in resistance between MRSA and MSSA are shown in Table 2. All isolates exhibited susceptibility to linezolid and vancomycin. Among the 63 *S. aureus* isolates, resistance rates were observed as follows: 79.4% to erythromycin, 66.7% to ciprofloxacin, 60.3% to cefoxitin, and 58.7% to levofloxacin. Clindamycin showed the least resistance. MRSA isolates exhibited markedly greater resistance than MSSA isolates for the following antibiotics: clindamycin, ciprofloxacin, ofloxacin, levofloxacin, tetracycline, and doxycycline.

### 3.3. MLSB Phenotypes and Biofilm Formation

Biofilm formation and MLSB phenotypes among *S. aureus* isolates are shown in Table 3. The D-test results indicated that constitutive macrolide–lincosamide–streptogramin B (cMLSB) resistance was demonstrated in 21 isolates (33.3%, 21/63), while inducible MLSB (iMLSB) resistance was observed in 17 cases (27%, 17/63). Macrolide–streptogramin B (MS) resistance accounted for 20.6% of isolates (13/63), while 19% (12/63) were sensitive. Biofilm production was detected in 76.2% of the isolates, with 30.2% classified as strong biofilm formers.

### 3.4. Correlation Between Biofilm Formation and Antibiotic Resistance

Isolates that produce biofilms exhibited significantly greater resistance to cefoxitin (70.8% compared to 26.7%, *p* = 0.002) and clindamycin (39.6% compared to 6.7%, *p* = 0.024). No significant differences in resistance to levofloxacin, ciprofloxacin, and gentamycin were observed between biofilm-producing and non-biofilm-producing isolates. (Table 4).

### 3.5. Characterization of intI1 Gene-Positive S. aureus Isolates

Class 1 integrons were the most prevalent and accounted for 33.3% (21/63), whereas the *intI2* or *intI3* were not detected. The *intI1* gene was detected in 55.3% (21/38) of MRSA isolates, while 44.7% (17/38) were negative for this gene. Additionally, the gene was not detected in any MSSA isolates. All *intI1* gene-positive isolates were MRSA and biofilm producers, with most categorized as strong biofilm formers. These isolates exhibited a multidrug-resistant pattern, with frequent resistance to cefoxitin, erythromycin, clindamycin, ciprofloxacin, ofloxacin, levofloxacin, tetracycline, doxycycline, and trimethoprim/sulfamethoxazole (Table 5).

### 3.6. Logistic Regression Analysis for Predictors of Biofilm Production

The multivariate logistic regression analysis showed significant correlation between biofilm formation and certain microbiological and genotypic factors, particularly the type of specimen: isolates from pus displayed significantly higher biofilm formation *p* = 0.02), while other specimen types (ETT, blood, sputum) did not reach statistical significance. Regarding the presence of *mec A* and *intI1* genes, the results show a significant correlation between biofilm formation and the presence of *mec A* gene, with biofilm producers having a much higher rate of this gene compared to non-producers (*p* = 0.049). *intI1*-positive isolates all produced biofilm (100%), preventing the calculation of an odds ratio (zero value for non-biofilm producers). In terms of MLSB phenotypes, cMLSB phenotypes were significantly associated with biofilm production *p* = 0.04) (Table 6).

## 4. Discussion

Over the past decade, MRSA infections have escalated to epidemic levels worldwide, creating considerable therapeutic difficulties due to the growing complexity of treatment. *S. aureus* is classified as a high priority for the discovery and production of novel antibiotics by the World Health Organization [22,23]. The current research investigated the prevalence of Class 1, 2, and 3 integrons, biofilm formation, and antimicrobial resistance patterns in clinical *Staphylococcus aureus* isolated from hospital-acquired infections in a tertiary care hospital in Egypt.

In this study, a total of 63 *S. aureus* strains were examined. Most strains were isolated from pus samples, followed by blood, with endotracheal aspirates being least frequent (14.3%). The prevalence rate of MRSA in the current study was 60.3%. Our findings agree with those of Zomorodi et al. and Mohammadi et al., who reported that the prevalence of MRSA was 59.6% and 59.1%, respectively [21,24]. Naimi and colleagues in Afghanistan, along with Dendi et al. in Algeria, reported that 56% and 50% of isolated *S. aureus* strains were MRSA, respectively [25,26].

Significant regional variations exist; for instance, Mahfouz et al. reported a substantially higher MRSA prevalence of 94.5% [27]. Additionally, El Maghraby et al. [28], Amr and Gammal [29], and Saeed et al. [30] found that MRSA accounted for 85%, 78%, and 76% of their *S. aureus* isolates, respectively. Moreover, studies from Nepal and Eritrea documented rates of 75% and 72%, respectively [31,32]. Variations in antibiotic prescribing practices, infection control measures, and healthcare infrastructure across regions may account for these discrepancies. Conversely, lower MRSA prevalence was reported in other Egyptian studies [33,34,35]. This indicates that there is variety even within a single nation, potentially due to variations in study populations or methodologies. Additionally, Jomehzadeh N. Emrani reported a lower prevalence, finding that 48.3% of the isolated *S. aureus* were MRSA [19], while Moghaddam et al. and Qodrati et al. observed that 39.4% and 37.5% of examined *S. aureus* isolates were MRSA, respectively [36,37]. These discrepancies may result from local factors, such as climate or microbe prevalence, while others are likely attributable to differing preventative techniques, topical and systemic treatments, sample regimens, and study durations [38].

Regarding the susceptibility pattern of *S. aureus,* the current investigation revealed that *S. aureus* isolates exhibited the highest resistance rates to erythromycin (79.4%), ciprofloxacin (66.7%), cefoxitin (60.3%), levofloxacin (58.7%), and tetracycline (55.6%). *S. aureus* isolates were resistant to doxycycline and ofloxacin (50.8% each), trimethoprim-sulfamethoxazole (44.4%), gentamicin (38.1%), and clindamycin (31.7%). However, these isolates were all sensitive to linezolid and vancomycin (100%).

In agreement with the findings of the present study, Zomorodi et al. reported that their isolates exhibited the highest resistance to erythromycin and ciprofloxacin, with frequencies of 86.3% and 66.1%, respectively. Furthermore, 59.6% were classified as MRSA. All isolates demonstrated susceptibility to linezolid [21].

In contrast, Adesoji et al. found that clinical *S. aureus* isolates were completely resistant (100%) to erythromycin but showed the least resistance to ofloxacin (27.5%) and gentamicin (20%) [39]. In addition, Kashef et al. [40] found that tested *S. aureus* isolates had a greater resistance rate to the fluoroquinolone class (76.3%). Staphylococci features and antimicrobial resistance profiles vary depending on geographical area and antibiotic use [41].

The current investigation revealed significant variations in susceptibility levels to commonly used antibiotics (clindamycin, ciprofloxacin, ofloxacin, levofloxacin, tetracycline, and doxycycline) between MRSA and MSSA isolates. The findings of the present investigation were analogous to those of La Vecchia et al. [42]. MRSA strains were more resistant to clindamycin, macrolides, quinolones, and tetracycline than were MSSA isolates.

Clindamycin is considered a viable option for the management of infections caused by *S. aureus* due to its oral and parenteral availability, 90% oral bioavailability, low cost, and excellent tissue penetration. It also can suppress the production of *S. aureus* toxins [43].

The D test results indicate that the cMLSB phenotype was the most common macrolide resistance mechanism, found in 33.3% of cases; the iMLSB phenotype followed at 27.0% and the MS phenotype at 20.6%, while 19.0% of the isolates were completely susceptible to MLSB antibiotics. These phenotypic patterns draw attention to the frequency of MLSB-mediated resistance mechanisms in the examined *S. aureus* isolates. Our results correspond with various reports indicating that cMLSB is the predominant macrolide resistance mechanism among macrolide-resistant *S. aureus* isolates [28,44,45,46,47].

A primary cause of antibiotic resistance is the defiant features of the biofilms formed by these bacteria [14]. The capacity of *S. aureus* to produce biofilms markedly enhances antibiotic resistance and persistent infections [48].

Biofilm formation was evaluated phenotypically using tissue culture plate assays. Among the analyzed isolates, 48 (76.2%) exhibited biofilm production. The level of biofilm formation was categorized as follows: 19 (30.2%) were classified as strong producers, 17 (27.0%) as moderate producers, and 12 (19.0%) as weak producers. These results are consistent with other investigations evaluating biofilm formation by *S. aureus* isolates [14,49], while other studies show a higher number of biofilm producers. For instance, Abdelraheem et al. [50], Karki et al. [51], El Maghraby et al. [28], and Bimanand et al. [52] determined biofilm in 81.6%, 86.3%, 94.5%, and 96%, respectively, of their isolates. Conversely, Nasr et al. found that 46% of *S. aureus* isolates produced biofilm [53].

The current study showed that *S. aureus* strains that produce biofilms were much more resistant to cefoxitin and clindamycin than those that do not produce biofilms. This correlation highlights the function of biofilms in augmenting antibiotic tolerance through reduced drug penetration and metabolic dormancy. This finding agrees with Jomehzadeh & Emrani, and Banerjee et al., who identified significant disparities in susceptibility rates to routinely utilized antibiotics between biofilm-forming and non-biofilm-forming bacteria [19,54].

Integrons are essential genetic elements that retain cassettes with antibiotic resistance genes, contributing significantly to multidrug resistance (MDR) and complicating treatment options, leading to deterioration of patient outcomes [11]. In the present investigation, 21/63 (33.3%) of the *S. aureus* isolates contained the *intI1* gene. This finding was lower than other findings in Iran, China, and Egypt, which found that the incidence of *intI1* was 39.6%, 42.5%, and 46.6% of clinical *S. aureus* isolates, respectively [55,56,57]. This geographical variation may result from differences in antibiotic prescription practices, infection control measures, or genetic characteristics of prevalent *S. aureus* strains. While the incidence of intI1 was low in the current study, nearly all *S. aureus* isolates possessed the *intI1* gene and were identified as MRSA and MDR, which underscores the significance of this relationship when constraining treatment options.

However, this frequency exceeded that documented by Mohammadi et al. and Deng et al., who found that 24.8% and 31.6% of tested *S. aureus* isolates carried class 1 integrons, respectively [24,58]. Conversely, Maratha et al. and Mostafa et al. reported that 71% and 72.6% of MRSA isolates harbored the *intI1* gene, respectively [11,59]. In contrast, a recent study in Iran found that only 7.6% of *S. aureus* isolates had the *intI1* gene. All tested *S. aureus* isolates in our investigation were negative for the *intI2* and i*ntI3* genes. This finding is consistent with previous reports [21,59]. However, in earlier studies, class 2 integrons were detected in 3.4% and 35.2% of isolates, respectively [11,57]. The variation in integron prevalence underscores the importance of integrating molecular surveillance of integrons into antibiotic resistance monitoring programs to enhance treatment regimens.

The results show a significant correlation between biofilm formation and the presence of *intI1* and *mecA* genes in *S. aureus* isolates, with biofilm producers having a much higher rate of these genes compared to non-producers (*p* = 0.002). This finding is well matched with those reported by Pozzi et al. and Aghmiyuni et al., who emphasized that the existence of *mecA* was linked with enhanced biofilm formation [60,61]. This agrees with research revealing a correlation between biofilm development and antibiotic resistance in *S. aureus*, frequently promoted by the *mecA* gene [62]. In line with our results, there was a significant correlation between class 1 integrons and biofilm formation [13]. This observation is consistent with existing literature indicating that biofilm communities can enhance horizontal gene transfer among bacteria because of their closely packed structure [63]. However, we emphasize that this association does not essentially indicate a causal link between *intI1* and biofilm development, as our study did not analytically evaluate any mechanistic connections. To justify these preliminary results and clarify the fundamental mechanisms, additional studies with more detailed molecular analysis are required.

However, isolates from pus displayed significantly higher biofilm formation *p* = 0.02), while other specimen types (ETT, blood, sputum) did not reach statistical significance. This finding suggests that biofilm formation is more likely caused by genetic variables than by the place of infection. These results underscore the need to focus on genetic variables in the management of biofilm-associated infections.

Our study has a few limitations. It is a single-center study with a small sample size, which limits the generalizability of our findings. More extensive genotyping would add significant epidemiological value. Future research should include gene cassette sequencing, SCCmec typing, and multilocus sequence analysis with multicenter studies to provide deeper epidemiological insights.

## 5. Conclusions

The prevalence of MRSA isolates is alarming. Furthermore, there is a rising prevalence of cMLSB and iMLSB resistance phenotypes. Most *S. aureus* strains with the *intI1* gene were MRSA and MDR. Ultimately, these results highlight the persistent issue of antimicrobial resistance and require a reassessment of approaches for treatment of *S. aureus* infections. Our findings further emphasize the need for strict infection control and prompt antimicrobial stewardship activities that can prevent the dissemination of resistant bacteria.

## Figures and Tables

**Table 1:** Comparison between MRSA and MSSA isolates according to demographic and clinical characteristics.

	MRSA (n = 38)	MSSA (n = 25)	Test of Sig.	*p*
Age (years)				
Min.–Max	18–70	27–68	t = 2.227 *	0.030 *
Mean ± SD	41.47 ± 14.62	49.24 ± 11.68		
Median (IQR)	41 (28–53)	51 (40–60)		
Sex				
Male	23 (60.5%)	13 (52.0%)	χ^2^= 0.448	0.503
Female	15 (39.5%)	12 (48.0%)		
Type of specimen				
Blood	7 (18.4%)	6 (24.0%)	χ^2^= 4.591	^MC^ *p* = 0.340
ETT	4 (10.5%)	5 (20.0%)		
Urine	7 (18.4%)	5 (20.0%)		
Pus	15 (39.5%)	4 (16.0%)		
Sputum	5 (13.2%)	5 (20.0%)		

ETT: Endotracheal tube. IQR: Interquartile range. SD: Standard deviation. t: Student *t*-test. χ^2^: Chi-squared test. MC: Monte Carlo *p*: *p* value for comparing between MRSA and MSSA *: Statistically significant at *p* ≤ 0.05.

**Table 2:** Antimicrobial resistance patterns among MRSA and MSSA clinical isolates.

	MRSA (n = 38)	MSSA (n = 25)	χ^2^	*p*
Antibiotics				
Cefoxitin (FOX)	38 (100.0%)	0 (0.0%)	63.000 *	<0.001 *
Linezolid (LZD)	0 (0.0%)	0 (0.0%)	–	–
Vancomycin	0 (0.0%)	0 (0.0%)	–	–
Erythromycin (ERY)	30 (78.9%)	20 (80.0%)	0.010	0.920
Clindamycin (CLD)	18 (47.4%)	2 (8.0%)	10.786 *	0.001 *
Ciprofloxacin (CIP)	29 (76.3%)	13 (52.0%)	4.012 *	0.045 *
Gentamycin (GEN)	17 (44.7%)	7 (28.0%)	1.791	0.181
Ofloxacin (OFX)	24 (63.2%)	8 (32.0%)	5.857 *	0.016 *
Levofloxacin (LEV)	27 (71.1%)	10 (40.0%)	5.999 *	0.014 *
Tetracycline (TET)	30 (78.9%)	5 (20.0%)	21.221 *	<0.001 *
Doxycycline (DO)	28 (73.7%)	4 (16.0%)	20.076 *	<0.001 *
Trimethoprim/sulfamethoxazole (SXT)	20 (52.6%)	8 (32.0%)	2.600	0.107

χ^2^: Chi-squared test *p*: *p* value for comparing between MRSA and MSSA *: Statistically significant at *p* ≤ 0.05.

**Table 3:** Distribution of biofilm production and MLSB phenotypes among *S. aureus* isolates.

	No. (%)
Biofilm	
Non-biofilm producer	15 (23.8%)
Biofilm producer	48 (76.2%)
Weak	12 (19.0%)
Moderate	17 (27.0%)
Strong	19 (30.2%)
MLSB phenotypes	
MS	13 (20.6%)
cMLSB	21 (33.3%)
iMLSB	17 (27.0%)
Sensitive	12 (19.0%)

**Table 4:** Correlation of antibiotic resistance with the biofilm production.

	Biofilm Production				χ^2^	*p*
	Non-Biofilm Producer (n = 15)		Biofilm Producer (n = 48)			
	Resistance	Sensitivity	Resistance	Sensitivity		
Antibiotic resistance						
Cefoxitin (FOX)	4 (26.7%)	11 (73.3%)	34 (70.8%)	14 (29.2%)	9.314 *	0.002 *
Erythromycin (ERY)	11 (73.3%)	4 (26.7%)	39 (81.3%)	9 (18.8%)	0.437	^FE^ *p* = 0.489
Clindamycin (CLD)	1 (6.7%)	14 (93.3%)	19 (39.6%)	29 (60.4%)	5.715 *	^FE^ *p* = 0.024 *
Ciprofloxacin (CIP)	10 (66.7%)	5 (33.3%)	32 (66.7%)	16 (33.3%)	0.00	1.000
Gentamycin (GEN)	3 (20.0%)	12 (80.0%)	21 (43.8%)	27 (56.3%)	2.734	0.098
Ofloxacin (OFX)	7 (46.7%)	8 (53.3%)	25 (52.1%)	23 (47.9%)	0.134	0.714
Levofloxacin (LEV)	9 (60.0%)	6 (40.0%)	28 (58.3%)	20 (41.7%)	0.013	0.909
Tetracycline (TET)	6 (40.0%)	9 (60.0%)	29 (60.4%)	19 (39.6%)	1.929	0.165
Doxycycline (DO)	6 (40.0%)	9 (60.0%)	26 (54.2%)	22 (45.8%)	0.918	0.338
Trimethoprim/sulfamethoxazole (SXT)	4 (26.7%)	11 (73.3%)	24 (50.0%)	24 (50.0%)	2.520	0.112

χ^2^: Chi-squared test ^FE^ *p*: Fisher’s exact test *p*-value * Statistically significant at *p* ≤ 0.05.

**Table 5:** Resistance profile, clinical source, and biofilm production among *intI1* gene-positive *S. aureus* isolates.

Isolate No.	Clinical Source	MLSB Phenotype	Biofilm Production	Antibiotic Resistance Profile
1	Pus	cMLSB	Strong	FOX, ERY, CLD, CIP, OFX, TET, DO, SXT
3	Blood	cMLSB	Strong	FOX, ERY, CLD, CIP, OFX, GEN, DO, SXT
5	Pus	cMLSB	Strong	FOX, ERY, CLD, CIP, GEN, DO, SXT
6	ETT	cMLSB	Strong	FOX, ERY, CLD, CIP, GEN, OFX, LEV, DO, SXT
8	Pus	cMLSB	Weak	FOX, ERY, CLD, CIP, GEN, OFX, LEV, DO, SXT
10	Urine	cMLSB	Moderate	FOX, ERY, CLD, LEV, DO, SXT
12	Pus	cMLSB	Strong	FOX, ERY, CLD, OFX, DO, SXT
13	Blood	cMLSB	Moderate	FOX, ERY, CLD, OFX, TET
15	Pus	cMLSB	Strong	FOX, ERY, CLD, CIP, GEN, LEV, OFX, TET, DO, SXT
20	Pus	cMLSB	Strong	FOX, ERY, CLD, CIP, GEN, OFX, TET, SXT
23	Urine	cMLSB	Moderate	FOX, ERY, CLD, TET, DO, SXT
27	Pus	cMLSB	Strong	FOX, ERY, CLD, CIP, OFX, LEV, TET, SXT
32	Pus	cMLSB	Weak	FOX, ERY, CLD, CIP, GEN, LEV, TET, SXT
39	Urine	MS	Strong	FOX, ERY, CIP, GEN, OFX, LEV, TET, DO, SXT
42	Urine	iMLSB	Moderate	FOX, ERY CIP, GEN, OFX, LEV, TET, DO, SXT
44	Sputum	iMLSB	Strong	FOX, ERY, CIP, GEN, OFX, LEV, TET, DO
47	ETT	MS	Strong	FOX, ERY, CIP, OFX, LEV, TET
50	Blood	iMLSB	Weak	FOX, CIP, LEV, TET, DO
54	Sputum	Sensitive	Weak	FOX, CIP, OFX, LEV, TET, DO
60	Pus	Sensitive	Strong	CIP
63	Blood	Sensitive	Strong	FOX, CIP, OFX, LEV, TET, DO

**Table 6:** Logistic regression analysis for predictors of biofilm production.

Variables	Biofilm Production		Bivariate Analysis		Logistic Regression Analysis *	
	Non-Biofilm Producer (n = 15)	Biofilm Producer (n = 48)	*p* Value	COR (95% CI)	*p* Value	AOR (95% CI)
**Type of specimen**						
Urine	6 (50%)	6 (50%)		Ref.		
ETT	3 (33.3%)	6 (66.7%)	0.4	2 (0.3–11.9)		
**Blood**	3 (23.1%)	10 (76.9%)	0.2	3.3 (0.6–18.5)		
**Pus**	2 (10.5%)	17 (89.5%)	0.02	8.5 (1.3–54.1)		
**Sputum**	1 (10%)	9 (90%)	0.07	9 (0.9–94.9)		
***intI1* gene**						
**Negative**	15 (35.7%)	27 (64.3%)	0.002^*^	Ref.		
**Positive**	0	21 (100%)		Undefined		
***mec* A gene**						
**Negative**	11 (44%)	14 (56%)	0.002 *	Ref.	0.049	Ref.
**Positive**	4 (10.5%)	34 (89.5%)		6.7 (1.8–24.6)		4.2 (1.009–17.4)
**MLSB phenotypes**						
MS	7 (53.8%)	6 (46.2%)		Ref.		Ref.
cMLSB	1 (4.8%)	20 (95.2%)	0.007	23.3 (2.4–229.3)	0.04	12.2 (1.1–133)
iMLSB	3 (17.6%)	14 (82.4%)	0.05	5.4 (1.04–28.5)	0.08	4.7 (0.8–26.2)
Sensitive	4 (33.3%)	8 (66.7%)	0.3	2.3 (0.5–11.8)	0.5	1.7 (0.3–9.6)
					Constant % correctly predicted Model ꭓ^2^, *p*-value	−0.6 76.2 16.2, 0.003

*: Factors included in regression analysis included type of specimen, *mecA*, and MLSB phenotypes. COR: Crude odds ratio, AOR: Adjusted odds ratio, CI: Confidence interval.

## Data Availability

Data can be obtained from the corresponding author upon request.

## Supplementary Materials

The following supporting information can be downloaded at: https://www.mdpi.com/article/10.3390/pathogens14070705/s1, Table S1: Primers Sequences, Amplicon Sizes, and Thermal Cycling Programs for *mecA, intI1*, *intI2*, and *intI3* Genes.
