# Supplementary material for: Study of Class 1, 2, and 3 Integrons, Antibiotic Resistance Patterns, and Biofilm Formation in Clinical Staphylococcus aureus Isolates from Hospital-Acquired Infections
Source: Pathogens. 2025 Jul 17;14(7):705. doi: 10.3390/pathogens14070705 (PMC12300923; doi:10.3390/pathogens14070705)
Supplement: Supplementary file 1 [file pathogens-14-00705-s001.zip › pathogens-3693078-supplementary.pdf]

Table S1. Primers Sequences, Amplicon Sizes, and Thermal Cycling Programs for *mecA*, *intI1*, *intI2*, and *intI3* Genes.

Target gene	Sequence (5-3′)	Amplicon Size	Denaturation Temperature	Annealing Temperature	Extension temperature	Reference
***mecA*-F**	GTAGAAATGACTGAACGTCCG ATAA	585 bp	95 ℃	55 °C	72 ℃	**[20]**
***mecA*-R**	CCAATTCCACATTGTTCGGTCT AA					
intI1-F	CCT CCC GCA CGATGATC	280 bp	95 ℃	58 ℃	72 ℃	**[21]**
intI1-R	TCCACGCATCGTCAGGC					
intI2-F	TTATTGCTGGGATTAGGC	233 bp				
intI2-R	ACGGCTACCCTCTGTTATC					
intI3-F	AGT GGGTGGCGAATGAGTG	600 bp				
intI3-R	TGTTCTTGT ATCGGCAGGTG					

BP: Base pair: F: Forward: R: Reverse
